# Genome wide analysis of the bovine mucin genes and their gastrointestinal transcription profile

**DOI:** 10.1186/1471-2164-12-140

**Published:** 2011-03-07

**Authors:** Prisca R Hoorens, Manuela Rinaldi, Robert W Li, Bruno Goddeeris, Edwin Claerebout, Jozef Vercruysse, Peter Geldhof

**Affiliations:** 1 Department of Virology, Parasitology and Immunology, Faculty of Veterinary Medicine, Ghent University, Salisburylaan 133, 9820 Merelbeke, Belgium; 2 Bovine Functional Genomics Laboratory, Animal and Natural Resources Institute, USDA-ARS, 10300 Baltimore Avenue, Beltsville, MD 20705, USA; 3 Department Biosystems, Division Gene Technology, Faculty of Bioscience Engineering, K.U. Leuven, Kasteelpark Arenberg 30, 3001 Leuven, Belgium

## Abstract

**Background:**

Mucins are large glycoproteins implicated in protection of all mucosal surfaces. In humans and rodents, the mucin gene family has been well described and previous studies have investigated the distribution and function of mucins in the gastrointestinal (GI) tract. In contrast, little data is available on the mucin gene family in polygastric species, such as cattle. The aim of the current study was to identify all members of the bovine mucin family by genome mining and subsequently investigate the transcription pattern of these mucins in the GI tract.

**Results:**

Nine bovine membrane-associated mucins (*MUC1*, *MUC3A*, *MUC4*, *MUC12*, *MUC13*, *MUC15*, *MUC16*, *MUC20 *and *MUC21*) and six secreted mucins (*MUC2*, *MUC5AC*, *MUC5B*, *MUC6*, *MUC7 *and *MUC19*) were identified in the bovine genome. No homologues could be identified for *MUC3B*, *MUC8 *and *MUC17*. In general, domain architecture of the membrane-associated mucins was found to be similar between humans and cattle, while the protein architecture of the gel-forming mucins appeared to be less conserved. Further analysis of the genomic organization indicated that the previously reported bovine submaxillary mucin (*BSM*) may be part of a larger gene encoding for MUC19. Analysis of the transcription profile showed that the secreted mucins were transcribed from the abomasum onwards, whereas the membrane associated mucins *MUC1 *and *MUC20 *were transcribed throughout the whole GI tract. In contrast to humans, *MUC5B *transcript was found in both the small and large intestine, but was absent in oesophageal tissue.

**Conclusions:**

This study provides the first characterization of the mucin gene family in cattle and their transcriptional regulation in the GI tract. The data presented in this paper will allow further studies of these proteins in the physiology of the GI tract in ruminants and their interactions with pathogens.

## Background

Mucins (MUC) are heavily O-glycosylated proteins that cover all mucosal surfaces. They play an important protective role as they form a physical, chemical and immunological barrier between the environment and the organism. Mucins can be largely divided into 2 structurally different families: the secreted and the membrane (cell surface)-associated mucins [1-4]. In humans, 7 members in the family of the secreted mucins have been identified, which can be further subdivided into gel-forming mucins (*MUC2, MUC5AC, MUC5B, MUC6, MUC19*) and non-gel-forming mucins (*MUC7, MUC8*) [5-7]. The membrane-associated mucins, on the other hand, count 11 members (*MUC1, MUC3A, MUC3B, MUC4, MUC12, MUC13, MUC15, MUC16, MUC17, MUC20 *and *MUC21*) [8]. Structurally, all mucins have at least one mucin-like domain, named PTS-domain, formed by a variable number of tandem repeats (VNTR) rich in Pro, Thr and Ser residues [9]. These PTS domains carry the typical mucin O-glycosylations [10]. The secreted gel-forming mucins are further characterized by the presence of other typical domains [11], such as N-terminal TIL domains, N- and/or C-terminal Von Willebrand D and C domains (VWD/VWC), C8 domains, and a C-terminal cystine knot (CT) domain, all involved in the oligomerization through the formation of disulphide bridges between cysteine residues [12,13]. The capacity to form oligomers gives the secreted gel-forming mucins the ability to build up the dense, visco-elastic mucus gel that covers many epithelia [4,14-17]. Secreted non-gel-forming mucins on the other hand are not able to oligomerize, and their structural and functional properties are not well described [18,19]. The cell surface-associated mucins also do not oligomerize, but they are characterized by specific domains such as the C-terminal sea urchin sperm protein-enterokinase-agrin (SEA) domain, cleaved after translation, an epidermal growth factor (EGF) or EGF-like (EGF-L) domain involved in the three dimensional structure, and a transmembrane domain (TM). MUC4, a member of the cell surface-associated mucins, together with the typical domains described above, contains a variant of the VWD domain that lacks cysteines, as well as an adhesion associated domain (AMOP) with a possible role in cell adhesion, and a NIDO domain of unknown function. The membrane bound mucins are mostly present on the apical membrane of epithelial cells, where they have been suggested to play a role in cell signalling. Some of them can also be found in the mucus layer, together with the secreted ones, probably due to proteolytic cleavage [20,21] or to the expression of secreted splicing variants [22,23].

Sequencing and annotation of mucin genes is known to be difficult due to the large size and repetitive structure of these molecules; moreover, among species, differences in the mucin gene family have been reported. Comparison between humans and mice, two mammals with a completed annotated genome [24], has shown that although the majority of mucins are commonly represented in both species, differences for few mucin genes are evident. In humans, for example, a *MUC17 *and two *MUC3 *genes (*MUC3A *and *MUC3B*) have been described, while in mice only one *MUC3 *gene has been identified and it is still under debate if it is the homologue of human *MUC3A *or *MUC17 *[25-27]. Moreover, the *MUC8 *gene has so far only been described in humans [28]. In ruminant species, such as cattle, only few mucin genes have been described to date (i.e. *MUC1 *[29-31], *MUC15 *[23,32] and bovine submaxillary mucin (*BSM*) [33,34]); therefore the first goal of the current study was to provide a comprehensive overview of annotated and non-annotated members of the bovine mucin family through database search and comparison with their potential human homologue. Since mucins play a fundamental role in the gastrointestinal (GI) tract defence mechanism, the distribution of the different mucins throughout the GI tract has been widely studied for monogastric mammalians [2,35,36]. However, in polygastric species, mucin distribution is still largely unclear. Therefore, the second goal of the current study was to investigate the transcriptional distribution of the identified bovine mucins throughout the GI tract of adult healthy cows.

## Methods

### Identification and classification of mucin genes

To identify mucin-encoding genes, the bovine genome assembly version Btau_4.0 was used, accessible through the NCBI database [37]. First, the database was term-searched for automatically annotated mucin genes. In a second step, mucin-encoding genes were also identified by BLASTn and BLASTp queries, using the nucleotide and amino acid sequences of 18 previously described human mucin molecules. These sequences included *MUC1*, *MUC2*, *MUC3A*, *MUC3B*, *MUC4*, *MUC5AC*, *MUC5B*, *MUC6*, *MUC7*, *MUC8*, *MUC12*, *MUC13*, *MUC15*, *MUC16*, *MUC17*, *MUC19*, *MUC20 *and *MUC21*. The predicted bovine mucins were subsequently aligned with their putative human homologue to calculate amino acid sequence similarity. This was done using the MegAlign software (DNAStar).

In the situation where multiple partial bovine sequences were found with either the same annotation or showing similarity to the same human mucin sequence, the respective genomic and mRNA sequences were examined with SeqMan software (DNAStar) for possible overlaps. In the case of such overlap, the sequences were assembled to build up a contiguous genomic and transcript sequence and subsequently manually curated to identify open reading frames and protein sequences. The curations were based on comparisons with the human genomic and mRNA sequences, BLAST searches and bovine EST sequences, as well as sequencing of the overlap after PCR on samples collected from bovine GI tissues. When no overlap was found, a PCR approach was used on cDNA samples in order to investigate whether the predicted partial mucin sequences belonged to the same gene or transcript. Amplified fragments were extracted from agarose gel with the NucleoSpin^® ^Extract kit (Macherey-Nagel), cloned into the pGEM^®^-T-Easy vector (Promega) and transformed to *Escherichia coli *DH5α cells (Invitrogen). Transformants were screened for inserts, according to the manufacturer's instructions and inserts were PCR-amplified with primers against the T7 and SP6 promoters located on the pGEM^®^-T-Easy vector. PCR products were cleaned-up and sequence-analyzed at the Centre for Medical Genetics, Belgium [38]. All the new sequences identified were then submitted to the EMBL database as mRNA direct entries or as experimental TPA (Third Party Annotation) entries.

### Protein structure analysis

The protein sequences of the human and bovine mucins were blasted against the SMART database [39] in the normal SMART mode, searching for Pfam domain and internal repeats. PTS regions were identified either by SMART as regions of low complexity [19] or by hand by calculating the percentage of P + T (>40%) and S (>5%) residues in the repeats. Protein sequences were analyzed for signal sequences using the SignalP 3.0 server [40].

### Sample collection

Tissue samples were obtained at the slaughter house from four healthy cows, i.e. two Holstein and two mixed breed animals (Belgian Blue White - Holstein). The animals selected were four years old with an average weight of 455 kg. Oesophagus, rumen, reticulum, omasum, abomasum (fundic and pyloric region), duodenum, jejunum, ileum, caecum, colon and rectum from the GI tract were included. Tissues were snap frozen on liquid nitrogen and stored at -80°C until RNA was extracted.

### RNA and genomic DNA extraction

Total RNA was extracted from tissue samples using the RNeasy Mini kit (Qiagen). To remove contaminating genomic DNA (gDNA), on-column DNase digestion was performed using the RNase-free DNase set (Qiagen) according to the manufacturer's instructions. RNA quality was verified using an Experion™Automated Electophoresis System (Bio-Rad), and concentrations were determined using a NanoDrop ND-1000 spectrophotometer (NanoDrop Technologies). For all samples, the RNA quality indicator (RQI) calculated by the Experion™ software (Bio-Rad) was >8.0, indicating high RNA integrity. Genomic DNA contamination was investigated with the SuperScript One-Step RT PCR kit (Invitrogen) using intron-spanning primers for glyceraldehyde-3-phosphate dehydrogenase (*GAPDH*) (Table 1). Genomic DNA was extracted from one tissue sample using the DNeasy Blood and Tissue Kit (Qiagen) as recommended by the manufacturer and it was used as a positive control for primer specificity in subsequent PCR reactions.

**Table 1:** Primer sequences and amplicon length

Gene	Forward Sequence	Reverse Sequence	AL^a ^(bp)
GAPDH^b^	GGGTCATCATCTCTGCACCT	GGTCATAAGTCCCTCCACGA	176
GAPDH	ACCCAGAAGACTGTGGATGG	CAACAGACACGTTGGGAGTG	178
ACTB	GACATCCGCAAGGACCTCTA	ACATCTGCTGGAAGGTGGAC	205
MUC1	CACTGCTGCCAGCCATATTA	TCAAACCCCAAATGCTTCTC	212
MUC2	CATGTGGAACCAGGAGGACT	ATGTTCCCAACCTCGACAGG	153
MUC3A	CCGTCACAAGTACATCTAACACAG	ACATATTCGAGGCGTTAGCA	150
MUC4	GGAGAGTGCAGAGTCCTTGG	AGCAGCAAAGCCAATGAAGT	323
MUC5AC	CAGACCCTCCACCTTCTTCA	GGTCCTCGAAGCTGTTCTTG	263
MUC5B	TCTACCTGACCGTGGAGACC	GTTGATGATGCTGCACTGCT	299
MUC6-1	CAGCAAGGACAAAATCGTGA	CTCTGGTCTGGCCTCTGAAC	218
MUC6-2	CACGGCCTCCTGTCTTCTAC	AGAAGATGGACTGGCTCTCA	190
MUC7	TCCTGCTCCTAAGGCTACCA	GTGGAGGGGAGTGGTACTGA	155
MUC12	TACAGGGGCAACAACTTTCC	CGTCTCATCGTAGCACAGGA	234
MUC13	ACCTGGGACAGAGACACCAC	GATTGTCGGGGTAGGAGACA	262
MUC15	CTGCCTTGGAACTCATCCAT	CACAGACGTGGTGTTTGGTC	183
MUC16	TTCAGAAACAGCAGCATTGG	GTGTAGTGGTCCAGCCGAGT	200
MUC19	TTCTGGAGGGGCTGAATATG	CTCTCGTCCACCAAAAGAGC	204
MUC20	GGACATCACTGCTCTGACGA	GGGCTATTGTCCAGGTCTCA	239
MUC21	CAACCACAGGAGGCTCTGAT	CAAAGAATCCCACGACCACT	173
BSM	GGAGACATATGGACTGCCAAT	CACAGTATGCAATTTCACAGCA	194

^a^AL: cDNA amplicon length

^b^GAPDH intron-spanning primers, used to investigate gDNA contamination

### cDNA synthesis and PCR

One μg of total RNA was converted to cDNA using the iScript cDNA synthesis kit (Bio-Rad), following the manufacturer's instructions. Primers used to perform the mucin PCRs are listed in Table 1 and were designed with an annealing temperature of 60°C using the Primer3 software [41]. PCR amplifications were carried out using the GoTaq^® ^Flexi DNA Polymerase kit (Promega). A total reaction volume of 25 μl was used, containing 1 μl of single-stranded cDNA (5 ng of the input total RNA equivalent); 0.4 μM forward primer; 0.4 μM reverse primer; 0.65 U GoTaq^® ^DNA Polymerase; 0.2 mM each dNTP (PCR Nucleotide Mix); 1.5 mM MgCl_2_, 1x Green GoTaq^® ^Flexi Buffer. The PCR reactions were cycled as followed: an initial denaturing step of 4 min at 95°C, followed by 35 cycles of 30s at 95°C, 30s at 60°C, 30 s at 72°C and a final extension of 10 min at 72°C. PCR products were analyzed by electrophoresis in 1.5% agarose (Promega, Madison) gel at a constant voltage of 110 V with 1X Tris-acetate EDTA (TAE) buffer. DNA was visualised by ethidium bromide and analyzed using Image Quant™ 350. Image background was subtracted using the rolling ball method and band volumes were normalized against the known volume of the ladder 500 bp band (100 bp ladder, Promega) in each gel. Values obtained were arbitrarily grouped and bands classified as absent (green), present weak (yellow) and present strong (red). To control the quality of the synthesized cDNA in all the samples and to validate the classification based on band intensity, PCR on two housekeeping genes, *GAPDH *and β-actin (*ACTB*), was also performed.

## Results

### Cell surface associated mucins

**Figure 1:**
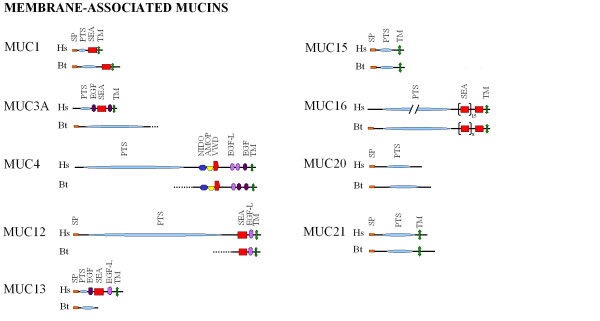
**Protein architecture of the bovine cell surface associated mucins**. Protein architecture of the human (Hs) and bovine (Bt) cell surface associated mucins MUC1, MUC3A, MUC4, MUC12, MUC13, MUC15, MUC16, MUC20 and MUC21. MUC16 is presented as a shortened sequence, indicated by //. Domains detected by SMART [39] are SP: signal peptide; PTS: Pro, Thr and Ser rich region; SEA: sea urchin sperm protein-enterokinase-agrin, TM: transmembrane domain; EGF: epidermal growth factor domain; EGF-L: EGF-like domain; NIDO: extracellular domain of unknown function; AMOP: domain involved in cell adhesion; VWD: Von Willebrand Factor D.

Searching the latest bovine genome assembly resulted in the identification of 10 sequences encoding 9 different cell surface associated mucins (Table 2). The percentage similarity, at amino acid level, between the predicted bovine mucins (bMUC) and their human homologues is reported in Table 2. A single bovine gDNA sequence encoding a predicted mRNA sequence was found for *MUC1*, *MUC3A*, *MUC4*, *MUC12*, *MUC13*, *MUC15*, *MUC20 *and *MUC21 *(Table 2). Two *MUC16*-like sequences were identified and no bovine homologous sequences were found for human (h)*MUC17 *and h*MUC3B*. Although all these bovine mucins showed low (<70%) similarity with their human homologues, the overall protein architecture was found to be conserved (Figure 1), with the exception of MUC3A, MUC12 and MUC13.

**Table 2:** Bovine and human mucin genes and protein similarity

Bos taurus				Homo sapiens				
**Gene**	**CDS**	**gDNA**	**mRNA**	**Gene**	**CDS**	**gDNA**	**mRNA**	**%**

**Membrane-associated mucins**								
*MUC1*	FL	NC_007301	NM_174115	*MUC1*	FL	NC_000001	NM_002456	62.4
*MUC3A*	3'p	NC_007326	XM_001255602	*MUC3A*	FL	NC_000007	XM_001725354	12.4
-				*MUC3B*	FL	AC_000050	XM_001125753	ND
*MUC4*	5'p	NC_007299	XM_001788490	*MUC4*	FL	NC_000003	XM_018406	66.6
*MUC12*	FL	NC_007326	BC142358	*MUC12*	FL	NC_000007	NM_001164462	68.2
*MUC13*	FL	NC_007299	XM_865756	*MUC13*	FL	NC_000003	NM_033049	19.6
*MUC15*	FL	NC_007313	NM_176631	*MUC15*	FL	NC_000011	NM_001135091	65.9
*MUC16*	FL	BN001315	BN001315*	*MUC16*	FL	NC_000019	NM_024690	57.6
-				*MUC17*	FL	NC_000007	NM_001040105	ND
*MUC20*	FL	NC_007299	XM_580797	*MUC20*	FL	NC_000003	NM_152673	35.5
*MUC21*	FL	NC_007324	XM_001255727	*MUC21*	FL	NC_000006	NM_001010909	38.4
**Secreted mucins**								
*MUC2*	FL	NW_001494549	BN001490*	*MUC2*	FL	NC_000011	NM_002457	44.8
		NW_001027928						
*MUC5AC*	FL	BN001491*	BN001491*	*MUC5AC*	FL	NC_000011	LT200503**	33.2
*MUC5B*	FL	BN001492*	BN001492*	*MUC5B*	FL	NC_000011	NM_002458	23.6
*MUC6-1*	3'p	NW_001027928	XM_867810	*MUC6*	FL	NC_000011	NM_005961	61.4
*MUC6-2*	3'p	NW_001494551	XM_870967	*MUC6*	FL	NC_000011	NM_005961	34.0
*MUC7*	3'&5'p	NW_001505809	XM_001255642	*MUC7*	FL	NC_000004	NM_152291	38.4
-				*MUC8*	5'p	-	U14383	ND
*MUC19*	5'p	BN001489*	BN001489*	*MUC19*	3'p	NC_000012	XM_002343162	64.8
*BSM*	5'p	NC_007303	XM_615336	*MUC19*	5'p	NC_000012	XM_002343163	50.7

CDS: coding sequence

gDNA: genomic DNA accession number in GenBank

mRNA: messenger RNA accession number in GenBank

%: percentage amino acid similarity between human and bovine mucin homologue

FL: full length

3'p: 3'partial, 5'p: 5'partial

ND: not determined

* EMBL accession number assigned to the newly generated sequences

** mRNA sequence retrieved from the Mucin Biology Group mucin database [71]

The predicted bMUC3A was found to contain a signal peptide (SP) and a PTS repeat region. However, the C-terminal EGF, SEA and TM domains, present in the hMUC3A protein sequence, were not found in the predicted sequence (Figure 1). Analysis of bMUC12 on the other hand, did show the presence of the C-terminal SEA, EGF-L and TM domains, as seen in hMUC12, but there was no N-terminal SP or PTS region (Figure 1). At genomic level, bMUC3A is located immediately upstream of bMUC12 on chromosome 25 and has the same orientation. However, there is gap in the genomic sequence of about 5 kb between both genes, explaining why the 3'end of bMUC3A and the 5'end of bMUC12 are still missing. For bMUC13, a SP and PTS repeat have been identified, but no other domains present in hMUC13 have been found. Although the bMUC13 nucleotide sequence (GenBank: XM_865756) was discontinued from the database, transcription of bMUC13 was evident in GI tissue samples. However, since bMUC13 has not been mapped on the current genome build Btau_4.0, no further analysis to identify a putative 3'end was performed.

Two partial b*MUC16 *mRNA (GenBank: XR_042722, GenBank: XM_001254224), together with their respective gDNA sequences (GenBank: NC_007305.4 and GenBank: NC_007305.2), were found in the database. Alignment of these sequences showed an overlapping region in both the gDNA and mRNA sequences, which was subsequently confirmed by PCR and sequencing. The new full-length gDNA and mRNA sequence was submitted to EMBL under accession number EMBL: BN001315 (Table 2). Comparison of the domain architecture of bMUC16 with the human homologue showed that the protein structure was largely conserved (Figure 1), with the exception of a signal peptide only found in the bovine sequence.

### Secreted mucins

Twelve bovine gDNA and predicted mRNA sequences were found in the sequence dataset, predicted to encode 7 secreted mucins (Table 2). Gel-forming *MUC2*, *MUC5AC*, *MUC5B*, *MUC6, MUC19*, *BSM *as well as the non gel-forming *MUC7 *were identified. No putative bovine homologue was found for human *MUC8 *(Table 2).

Two partial bovine *MUC2*-like mRNA sequences (GenBank: XM_598117 and GenBank: XM_001256289), together with their corresponding gDNA (GenBank: NW_001027928 and GenBank: NW_001494549), were found in the database. Alignment with the human *MUC2 *homologue showed that one b*MUC2*-like mRNA sequence (GenBank: XM_598117) aligned to the 5'end of h*MUC2 *and the other one (GenBank: XM_001256289) to the 3'end, with no overlapping region. In an attempt to identify and sequence the missing central part of bovine *MUC2 *mRNA, a PCR was performed on cDNA samples of the colon using a primer set spanning the missing region. The amplicon obtained in the PCR was subsequently cloned and sequenced. The two predicted sequences and the newly experimentally identified sequence were aligned and used to compose a new full-length b*MUC2 *mRNA sequence that was submitted to EMBL under the accession number BN001490 (Table 2). At genomic level, the same approach was taken but without success. Further blast search of the mRNA sequence against the bovine genome did not result in the identification of this region; therefore the gDNA sequence encoding the identified central part of the *MUC2 *mRNA still remains unknown.

Two predicted b*MUC5*-like sequences (GenBank: XM_604045 and GenBank: XR_042814) were found in the database. Sequence analysis indicated that the mRNA sequence XM_604045 showed the highest similarity to h*MUC5AC *and was therefore annotated as b*MUC5AC *in the EMBL database (EMBL:BN001491) (Table 2). The predicted mRNA sequence with GenBank accession number XR_042814 showed the highest similarity to h*MUC5B*. At genomic level, b*MUC5AC *and b*MUC5B *sequences were found to overlap, indicating that the b*MUC5AC *and b*MUC5B *genes are located in a cluster on chromosome 29. According to the database, exons 1 to 7 of the predicted b*MUC5B *mRNA sequence were identical to exons 4 to 10 of the predicted b*MUC5AC *mRNA. To investigate whether b*MUC5AC *and bMUC5B are indeed alternatively spliced, or whether this has been wrongly annotated, the 5' end of the b*MUC5B *genomic sequence was analyzed for putative exons using the GENSCAN server [42]. Eight putative exons were identified encoding *MUC5B *like sequences. Transcription of this newly identified b*MUC5B *5'end was experimentally shown by PCR amplification on cDNA. The corrected b*MUC5B *mRNA sequence has now been submitted under accession number BN001492 (Table 2).

**Figure 2:**
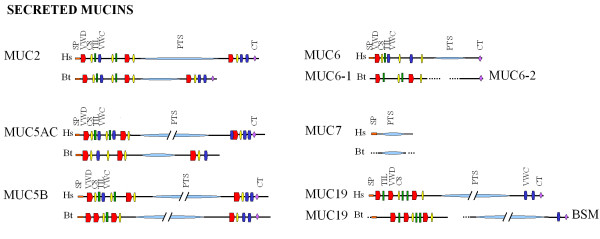
**Protein architecture of bovine secreted mucins**. Protein architecture of the human (Hs) and bovine (Bt) secreted mucins MUC2, MUC5AC, MUC5B, MUC6, MUC7 and MUC19. MUC5AC, MUC5B and MUC19 are presented as a shortened sequence, indicated by //. Domains detected by SMART [39] are SP: signal peptide, VWD: Von Willebrand Factor D; C8: domain with 8 conserved Cys residues; TIL: domain typically containing 10 Cys residues; VWC: Von Willebrand Factor C; PTS: Pro, Thr and Ser rich region; CT: C-terminal cystine knot-like domain.

At protein level, the predicted bMUC5AC showed an overall similarity of 33.2% with the human homologue, although the similarity increases if comparing the N-terminal region (amino acid AA 1-1350) before the PTS repeats (70.3%) and the C-terminal region (AA 2800-3554) after the PTS repeats (58.3%) (Figure 2). Similarly, bMUC5B showed a low overall similarity (23.6%) with the human homologue, but 75.2% and 53.2% similarity at the N-terminal region (AA 1-1040), and the C-terminal region (AA 5800-6725) respectively (Figure 2). Some domains of the hMUC5AC and hMUC5B protein are not found in bMUC5AC and bMUC5B (Figure 2). hMUC5AC has an N-terminal SP, VWD, C8, TIL and VWC domain (AA 1-443), of which TIL is not present in bMUC5AC (AA 1-459). These domains are followed by a second C8, TIL and VWC domain in hMUC5AC (AA 624-827), while only the second C8 domain in this region is found in bMUC5AC. A second VWD and third C8 domain before the PTS region are present both in hMUC5AC (AA 892-1162) and bMUC5AC (AA 893-1161). At the C-terminus of hMUC5AC, a VWC, VWD, C8, a second and third VWC and CT domain have been found, but in bMUC5AC, only the VWD, C8 and a VWC corresponding to the third VWC in hMUC5AC have been identified. Likewise, the organization of the domains is different for hMUC5B and bMUC5B. While hMUC5B contains the typical N-terminal SP, VWD, C8, TIL, VWC domain organization (AA 1-458), only the SP and VWD (AA 1- 216) have been conserved in bMUC5B, while the C8, TIL and VWC from hMUC5B have been replaced with a second VWD in bMUC5B (AA 234-401). The domain organization of the remaining protein sequence is similar between hMUC5B and bMUC5B.

Two partial mRNA sequences were found in the database for *MUC6*. One bovine sequence (bMUC6-1; GenBank: NW_001027928) showed to align to the 5' end of h*MUC6 *and one (bMUC6-2; GenBank: NW_001494551) to the 3'end. For both *MUC6*-like sequences corresponding gDNA sequences were also identified in the database. The alignment of all the bovine sequences found did not show any overlap at mRNA or gDNA level. Therefore, an attempt to identify the missing central region was done using a PCR approach, but without success. Although the similarity between hMUC6 and the N-terminal part of bovine MUC6-1 (AA 1-1170) is 70.8%, the domain organization was found to be different (Figure 2). In humans, SP, VWC, C8, TIL and VWC domains (AA 1-427) are present in the N-terminal region followed by C8, VWC and C8 domains. Instead, in bovine MUC6-1 only a VWD and a TIL domain are present in the N-terminal region followed by C8, TIL and VWD domains. In the central part of hMUC6 there is a PTS repeat region, which is still not identified in cattle (Figure 2).

Three partial sequences with similarity to human *MUC19 *were identified in GenBank. Two sequences (GenBank: XM_001788043 - similar to submaxillary apomucin, GenBank: XM_603306 - similar to *MUC19*) were found to align to the 5' end of h*MUC19*. Alignment of these two sequences showed an overlap at both the mRNA and gDNA level; hence a longer 5' partial b*MUC19 *(gDNA and mRNA) was constructed and submitted to the database under accession number BN001489. The third sequence identified in the database was the one encoding for bovine submaxillary mucin (BSM, GenBank: XM_615336). BSM is one of the few bovine mucin previously described [33,34,43] and it was found to have a high similarity (74% on amino acid level) with the C-terminal part of hMUC19 at protein level. The predicted *bMUC19 *and *BSM *genes are both on chromosome 5 in cattle, separated by about 9 kb of genomic sequence, suggesting that *BSM *together with the predicted *bMUC19 *might belong together and form one unique *MUC19 *gene. To confirm this hypothesis the genomic sequence between *BSM *and *bMUC19 *was analyzed using the GENSCAN server [42]. Only short exons (21-167 bp, at suboptimal cut-off level 0.01) could be predicted in this region and none of them encoded for mucin domains or PTS-rich peptides. Also in humans the central PTS region of *MUC19 *is unknown, but a putative full-length *hMUC19 *has been proposed by Chen *et al. *(2004) [44] (Figure 2). On the other hand in mice and in pigs full-length genes homologous to *hMUC19 *have been previously identified, named *Muc19 *and porcine submaxillary mucin (*PSM*), respectively. At protein level, alignment of both bMUC19 and BSM with human, mouse and pig MUC19 suggests that all the expected domains, typical for MUC19, are represented in bMUC19 and BSM. Assuming that the complete bovine MUC19 has a similar length compared to PSM and mouse Muc19, it would mean that the unknown region between BSM and bMUC19 is about 500 AA long. For this reason PCR was performed on jejunal samples in an attempt to identify this putative linkage region. However, no amplification was obtained, likely due to the presence of a repetitive sequence encoding the PTS-rich regions.

### Membrane associated and secreted mucins are differentially transcribed in the gastrointestinal tract

**Figure 3:**
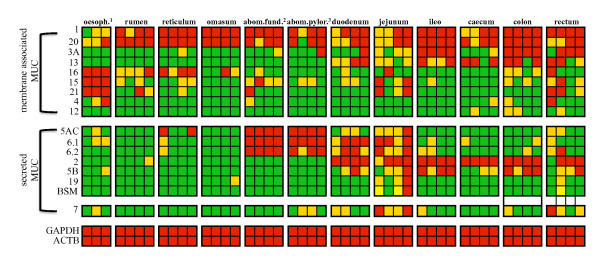
**Transcription profile of bovine mucins in the gastrointestinal tract**. Transcription profile of membrane associated and secreted mucins in the gastro-intestinal tract. PCR on cDNA samples was used to characterize the transcription profile of membrane associated and secreted mucins in twelve regions of the gastro-intestinal tract. Squares represent PCR products of individual animals (*n *= 3 in oesophagus; *n *= 4 in all other tissues). Presence and intensity of gel bands were analyzed using ImageQuant™350 and are reported as absent (green), present weak (yellow), and present strong (red). Glyceraldehyde 3-phosphate (GAPDH) and β-actin (ACTB) were used to control the quality of the synthesized cDNA in all the samples.

To understand the transcription profile for all the known bovine mucins in the gastro-intestinal tract, PCR was performed on cDNA of samples collected from several tissues. The results obtained showed a characteristic transcription profile for many of the mucins analyzed in both the membrane associated and the gel-forming groups (Figure 3). Among the membrane associated mucins, *MUC1 *and *MUC20 *were majorly transcribed, with occurrence of bands in all the tissues examined. *MUC15*, *MUC16*, and *MUC21 *were found to be transcribed in the first part of the GI tract with the strongest presence in the oesophagus of all the animals analyzed, while *MUC3A *and *MUC13 *were transcribed in the small and large intestine only. In contrast, *MUC12 *was hardly present in any of the tissues analyzed with only weak bands in few samples collected from the large intestine. *MUC4 *transcription was only detected in two samples collected in the oesophagus and one in the fundic region of the abomasum, caecum, colon and rectum.

In regard to the secreted gel-forming mucins, *MUC5AC *and *MUC6 *(both *MUC6-1 *and *MUC6-2*) were found to be consistently transcribed in the abomasum, both fundic and pyloric regions, and in the first part of the intestinal tract (duodenum and jejunum). *MUC2 *and *MUC5B *were only transcribed in the intestinal section of the GI tract with bands present in the majority of the tissues collected from this area. *MUC19 *and *BSM *showed a similar transcription pattern, with weak to strong bands in 3 animals in the jejunum and weak transcription in one sample in the rectum. *MUC19 *was also found to be weakly transcribed in the omasum of one animal, whereas the *BSM *transcript was not detectable in this sample. The secreted non gel-forming mucin *MUC7 *was transcribed in a limited number of tissues analyzed. Finally, for the two control genes, *GAPDH *and *ACTB*, strong bands were obtained in all the samples analyzed.

## Discussion

The current study identified 15 bovine mucin-encoding genes in the cattle genome (assembly Btau_4.0), including nine membrane-associated mucins and six secreted ones. Compared to human, no homologues could be identified for *MUC3B*, *MUC8 *and *MUC17*. This feature is not unique to cattle since previous studies showed that these three mucins were also absent in rodents [25,27]. In humans, the two genes encoding *MUC3A *and *MUC3B *show a high identity at nucleotide level both in the intronic and exonic sequences, suggesting that they are the product of a rather recent gene duplication after the divergence of humans and rodents [25]. This hypothesis would explain why *MUC3A *and *MUC3B *are found in chimpanzee, which diverged from humans later compared to rodents, but not in cows and other ruminants which diverged from humans earlier than rodents [45]. In addition, high similarity among human *MUC17 *and rodent *MUC3 *has previously been shown [27]. Although it has previously been suggested that human *MUC17 *is the true structural homologue of rodent *MUC3 *[27], the conserved localization of the *MUC3 *gene upstream of *MUC12 *and *MUC17 *in humans and upstream of *MUC12 *in mice and cattle, suggests that *MUC3 *in the latter two species is the homologue of human *MUC3 *rather than of *MUC17*. In regard to *MUC8*, BLAST analysis suggested that humans are the only species having this gene in their genome (data not shown).

As previously stated, sequencing mucin genes is technically difficult due to their size and the large number of repetitive sequences in the PTS regions. Therefore, some of the mucin gene and mRNA sequences are still incomplete. For example for *MUC6*, sequence information for the central PTS region is still missing. Similar problems were encountered for *MUC3A*, *MUC7, MUC12 *and *MUC13*.

In humans, two clusters of mucins have been previously described. *MUC3*, *MUC12 *and *MUC17*, encoding membrane bound mucins, are clustered on human chromosome 7q22 [27], while *MUC6*, *MUC2*, *MUC5AC*, and *MUC5B*, encoding secreted gel-forming mucins, are clustered on human chromosome 11p15 [46]. With the exception of MUC17, for which no bovine homologue has been identified, both gene clusters appear to be maintained in cattle, on bovine chromosome BTA25 and BTA29, respectively. This conservation has also been described for the *MUC6*, *MUC2*, *MUC5AC *and *MUC5B *gene cluster in mice [36].

Alignment of the bovine and human sequences showed that, although the characteristic regions and domains of mucin glycoproteins are generally conserved between these species, the overall sequence similarity is very low with values included between 16.9% and 68.2%. The reason for this low similarity is probably related to the presence of the PTS regions, which are characteristic of these proteins [47]. This is consistent with previous studies that compared human and rodent mucins [26,48,49]. In general, domain architecture of the membrane-associated mucins was found to be similar between humans and cattle, while the protein architecture of the gel-forming mucins appeared to be less conserved. The bovine MUC5AC, MUC5B, and MUC6 proteins did not show the VWD-C8-TIL-VWC domain organization which is typical for the N-terminus of all human secreted gel-forming mucins. Bovine MUC5AC contained no TIL domains over its entire length, while bMUC6 was shown to have no VWC domains. However, all the domains responsible for the oligomerization of these molecules, such as VWD and cystein rich domains, are present. In view of the sequence variation detected compared to the human *MUC *genes, it is still unclear what the variation is in the *MUC *genes of different cattle breeds. High variability in the number of VNTRs and the extent of VNTR polymorphism within and across cattle breeds has only been reported for the *MUC1 *gene [50,51]. The level of allelic variance for the other bovine *MUC *genes is still unclear.

*BSM *is one of the few mucins previously isolated and studied in cattle [33,34,43]. It is located on chromosome 5 and high sequence similarities have been shown with the C-terminal part of porcine and ovine submaxillary mucins (*PSM *and *OSM*). In 2004 [44,52], human and mouse *MUC19 *were reported as new members of the gel-forming mucin family. Their sequence similarity, similar expression pattern (majorly in salivary glands), and chromosomal location compared to *BSM *and *PSM *suggested that they were potential homologues. The previously reported BSM sequence lacked the N-terminal conserved protein domains present in PSM and other MUC19 sequences [34], suggesting that it was not complete. In the current study, a new mRNA and corresponding gDNA sequence (b*MUC19*) was identified showing homology to the 5' part of h*MUC19*. This gDNA sequence is located approximately 9 kb upstream of the *BSM *gene on chromosome 5. The genomic localization and the almost identical transcription profile of both genes would suggest that *BSM *and this newly identified b*MUC19 *are actually part of one gene encoding the bovine *MUC19 *homologue.

At the moment, mechanisms regulating human mucin transcription are largely unknown and promotor sequences have not been identified for all human *MUC *genes. The transcription factors NF-kappaB and AP-1 seem to be involved in the regulation of a number of human *MUC *genes, including the membrane bound mucin *MUC1 *[53] and the secreted mucins *MUC2 *[54-56], *MUC5AC *[57-59], *MUC5B *[60,61] and *MUC6 *[62]. In cattle, promotor sequences are available only for *MUC1*, *MUC3A*, *MUC5B*, *MUC12*, *MUC15*, *MUC20 *and *MUC21*. *In silico *promotor analysis showed potential binding sites for AP-1 in the secreted mucin *MUC5B *and for AP-1 and c-Rel/NF-kappaB in all the membrane bound mucins, with the exception of *MUC1 *(data not shown). As more sequence data become available, it will be interesting to investigate if different regulatory mechanisms exist within the different classes of mucin genes and whether they can affect (patho) physiological conditions.

Transcriptional analysis of the 15 identified mucin genes showed that most of them are transcribed in the GI tract of cattle. Although some individual differences were observed, in general, for the membrane associated mucins, *MUC1 *and *MUC20 *transcripts were detected in all the tissues examined, whereas *MUC3 *seemed to be mainly produced in the intestinal tissues. This is consistent with the observations made in humans [35,63]. *MUC15*, *MUC16 *and *MUC21*, on the other hand, were the major membrane-associated mucins found in the oesophagus. A search of the human EST dataset revealed the presence of *MUC15 *and *MUC21 *encoding ESTs derived from an oesophageal cDNA library (data not shown), suggesting that their transcription pattern might be conserved. Concerning the secreted mucins, *MUC5AC *and *MUC6 *were primarily transcribed in the abomasum, whereas *MUC2 *and *MUC5B *were the main secreted mucins in the intestinal tissues. This is largely in agreement with the situation in humans, except for *MUC5B*, for which evidence in the human intestine has only been reported in colonic tissue [64,65]. In contrast to our results in cattle, the human *MUC5B *is also found in the oesophagus, where it is transcribed in the oesophageal submucosal glands (SMGs). SMGs are known to differ in distribution and structures among species [66], so it is possible that the oesophageal samples collected in this study did not include regions rich in these glands. Alternatively, it is also possible that in cattle SMGs do not produce MUC5B. Interestingly, secreted mucins were not found to be produced in the pre-stomachs. Since secreted gel-forming mucins are widely acknowledged as an important component in the formation of viscoelastic mucus in stomach and intestine to protect the mucosal epithelium against acidic and proteolytic damages [67], the absence of gastric juices, hydrochloric acid and digestive enzymes in the pre-stomach may explain this lack of expression. On the other hand, the pattern of gene expression of both the membrane bound and secreted mucins in the pre-stomachs was very similar to the pattern observed in the oesophagus, which could be due to the common origin that these organs have during bovine embryogenesis [68]. Although the transcription pattern of bovine mucins in the GI tract is genererally similar to what is observed in humans, transcription levels do not always correspond with protein levels and different factors can influence the functional properties of the mature protein. Alternative splicing can generate different isoforms with different tissue distributions and properties. For several human membrane associated mucins, multiple isoforms have been identified, some of which are secreted [22,23,69]. In addition, posttranslational proteolysis of the SEA domain can also release the extracellular domain of membrane bound mucins into the mucus layer [20,29]. Since the large PTS regions constitute O-glycosylation sites, variance in the number, length and sequence of the repeats can impact the extent and type of glycosylation and thus the biological functions of mucins that are largely defined by their carbohydrate constitution [4]. Only a limited amount of studies have been performed to investigate these aspects in bovine mucins [50,70] and future studies will need to include these different factors influencing mucin biology.

## Conclusions

This study provides the first characterization of the mucin gene family in cattle and their transcriptional distribution in the GI tract. Homologues were identified for all members of the human mucin family, with the exception of human *MUC3B*, *MUC8 *and *MUC17*. The protein domain architecture of the membrane-associated mucins was found to be conserved between humans and cattle, while protein architecture of the gel-forming mucins appeared to be less conserved. Analysis of the transcription profile showed that the secreted mucins were transcribed from the abomasum onwards, whereas the membrane associated mucins *MUC1 *and *MUC20 *were transcribed throughout the whole GI tract.

## Authors' contributions

This article is a part of PH's PhD thesis. MR helped in performing the transcription studies. RL contributed to the genome mining. BG, EC, JV and PG conceived and designed the project. All authors read and approved the final manuscript.
